# Native parasite affecting an introduced host in aquaculture: cardiac henneguyosis in the red seabream *Pagrus major* Temminck & Schlegel (Perciformes: Sparidae) caused by *Henneguya aegea* n. sp. (Myxosporea: Myxobolidae)

**DOI:** 10.1186/s13071-020-3888-7

**Published:** 2020-01-14

**Authors:** Pantelis Katharios, Panos Varvarigos, Kleoniki Keklikoglou, Maja Ruetten, Jerry Sojan, Morgina Akter, Maria Chiara Cascarano, Maria Ioanna Tsertou, Constantina Kokkari

**Affiliations:** 1 Institute of Marine Biology, Biotechnology and Aquaculture, Hellenic Centre for Marine Research, Gournes, Heraklion, Greece; 2 Vetcare, Dimitressa Street, 115 28 Athens, Greece; 3 PathoVet AG, Buckstreet 2, CH-8317 Tagelswangen, Switzerland; 4 Department of Biology, University of Crete, 70013 Heraklion, Greece; 5 Scottish Association for Marine Science, University of Highlands and Islands, Oban, PA37 1QA UK

**Keywords:** *Henneguya aegea* n. sp., *Pagrus major*, Aquaculture, Exotic species, Parasite, Myxosporea

## Abstract

**Background:**

*Henneguya* Thélohan, 1892 (Myxobolidae) is one of the most species-rich genera of myxosporean parasites infecting fish. Although common in nature, there are few reports of these parasites causing important disease in aquaculture. In this paper, we describe a new species of *Henneguya* infecting *Pagrus major* (Temminck & Schlegel), a fish host introduced to the Mediterranean Sea from Japan in the late 1980s.

**Results:**

Large plasmodia of the parasite were found in the bulbus arteriosus and in the ventricle of the infected fish. Spores were found mainly in the kidney and heart and were accompanied by melanized macrophages or vascular intimal proliferation mixed with a mild non-suppurative response, respectively. Comparisons of morphometric data for spore and polar capsule length and width, suggest a unique combination of features in the newly described species. Molecular analysis, based on *18S* rDNA sequence of the parasite, followed by phylogenetic analysis, indicated that the parasite described here is a novel species of *Henneguya*, clustered with the marine congeneric species.

**Conclusions:**

*Henneguya aegea* n. sp. infects in aquaculture *P. major*, a host introduced as eggs to the Mediterranean from Japan. Despite the high host specificity of the myxobolid parasites, *H. aegea* n. sp. seems to be able to use *P. major* as a host and propagate successfully, causing morbidity and mortality. This could result in spillback of the new species from high density cultured non-native *P. major* to native fish hosts.

## Background

Since its inception, Mediterranean marine aquaculture has been mainly reliant on the farming of two fish species, the gilthead seabream, *Sparus aurata* Linnaeus, and European seabass, *Dicentrarchus labrax* (Linnaeus). Diversification, with the introduction of new species, is considered as the most appropriate way to ensure continued economic viability and sustainable growth of the industry in order to meet the increasing demand of consumers for high quality protein. Amongst the species that have long been considered as promising alternatives is the red porgy, *Pagrus pagrus* (Linnaeus), which is a highly valued, indigenous sparid [1]. Despite its good performance as an aquaculture species at all stages of production, the skin color of farmed red porgies darkens soon post-harvest while consumers expect the normal bright pink-red of the wild fish [1–3]. Thus, it was replaced in due course by the non-indigenous but morphologically similar congeneric red seabream, *Pagrus major* (Temminck & Schlegel) [4]. Being exotic to the Mediterranean Sea, the red seabream showed limited, if any, pathological problems related to parasites, at least at the beginning of its rearing history, consistent with the assumption that most locally occurring parasites will be specific for local indigenous hosts. On the other hand, as it has been described for salmonids cultured in net pens in British Columbia, naïve species may contract some unusual infections when placed in a new geographical area [5]. Many of these “unusual” infections in salmonids were caused by myxosporean parasites.

Myxosporeans are microscopic metazoan parasites mainly of fish, which belong to the class of Myxosporea of the phylum Cnidaria. They can be highly host-specific and, in many cases, they show organ- or tissue tropism. Many of these parasites have been reported to be highly pathogenic resulting in debility, reduced fecundity or mortality in their fish hosts [6]. Although myxozoan parasite infections are frequently observed in Mediterranean marine aquaculture [7], very few have been associated with severe epizootics. The most important pathogen of aquaculture fish is *Enteromyxum leei* (Diamant, Lom & Dykovà, 1994), which is responsible for severe losses in sparids, including the gilthead seabream, sharpsnout seabream (*Diplodus puntazzo* (Walbaum)) and red porgy, and unlike the majority of myxosporeans, has a wide host range [8, 9]. Another important parasite is *Sphaerospora testicularis* Sitjà-Bobadilla & Alvarez-Pellitero, 1990 which infects European seabass testes resulting in “parasitic castration” of the fish [10].

The genus *Henneguya* Thélohan, 1892 includes approximately 200 species described from all parts of the world, targeting mostly freshwater fish hosts and with a few also parasitic to marine fishes [11, 12]. *Henneguya* spp. are histozoic myxosporeans that infect several organs of fish including the gills and the heart. In 2005, *Henneguya pagri* Yokoyama, Itoh & Tanaka, 2005 was reported in red seabream farms of Japan as the causative agent of cardiac henneguyosis [13]. In the Mediterranean, *Henneguya* spp. have been reported in reared gilthead seabream from southern Italy, causing both morbidity and mortality [14], and from wild gilthead seabream caught off the coast of Tunis [15].

In this study, we describe a *Henneguya* sp. infection in cultured red seabream from the Aegean island of Leros, Greece. We suspect the parasite was acquired from local wild or cultured sparids after red seabreams were introduced to the Mediterranean, because these fish were imported as eggs from Japan in the 1980s thus precluding importation of myxosporeans from that region. We report morphological, histopathological and molecular analyses of this new parasite in an attempt to decipher its origin and possible sources of infection.

## Methods

Twenty apparently healthy fish (weight: 53.7–361.7 g, total length: 15.3–25.5 cm) were randomly collected in 2016 from the affected farm, which has been reporting persistent morbidity and mortality of cultured red seabream since 2010. The affected fish were reared in net pens in the sea. Usually, mortalities were in the range of 1–5% in each mortality episode and affected mostly larger fish. The fish were examined macroscopically for external and internal lesions or abnormalities. Samples from gills, kidney, liver and heart were fixed in 10% buffered formalin for routine histology. Infected hearts were dissected, and cysts of parasites were removed and fixed for electron microscopy in 2.5% glutaraldehyde in cacodylate buffer or examined fresh using a light microscope equipped with a digital camera.

### Spore morphology

*Henneguya* spores were obtained from freshly ruptured cysts and heart scrapings from the sampled fish and measured with the aid of a light microscope equipped with an image analysis system, calibrated with a micrometric scale, following the guidelines of Lom & Arthur [16]. Measurements were based on 55 fresh spores and are presented in micrometres as the range followed by the mean ± standard deviation, SD.

### Scanning electron microscopy (SEM)

Samples for SEM were washed with sodium cacodylate buffer, post-fixed with 1% OsO_4_ and dehydrated in an ascending alcohol series, mounted on stubs, sputter-coated with gold palladium and examined using a JEOL JSM-6390LV scanning electronic microscope at 15 kV at the Electron Microscopy Laboratory of the University of Crete.

### Histology

Formalin-fixed tissues were dehydrated in a 70–96% ethanol series and embedded in glycol methacrylate resin (Technovit 7100, Heraeus Kulzer, Germany). Serial sections (3–5 µm thick) were obtained using a microtome (Leica RM2245, Germany) with disposable blades. After drying, slides were stained with methylene blue/azure II/basic fuchsin (Polychrome) [17]. Another set of samples was also processed for paraffin sectioning; these tissues were fixed for 72 h in 10% buffered formalin, dehydrated in an ascending ethanol series (70–100%), embedded in paraffin and cut in 2–3 µm thin sections. They were stained using a standard protocol with haematoxylin and eosin (H&E).

### Micro-CT

Two infected and one uninfected heart were also examined with micro-CT. These samples were fixed in 4% phosphate-buffered formalin and dehydrated to 70% ethanol for 3 days before scanning. Subsequently, two different staining agents were used in each infected sample in order to enhance the contrast between the soft tissues. One affected and one control heart were stained with 0.3% phosphotungstic acid (PTA) in 70% ethanol while the second affected heart was stained with 1% iodine in 96% ethanol according to the protocol of Metscher [18]. The micro-CT scans of the hearts were performed at the Hellenic Centre for Marine Research (HCMR) using the SkyScan 1172 micro-CT scanner (SkyScan, Bruker, Belgium). This scanner uses a tungsten X-ray source with an anode voltage ranging from 20 to 100 kV, 11 MP CCD camera (4000 × 2672 pixel) and a maximal resolution of < 0.8 μm/pixel. The hearts #1 and the healthy control were scanned at a voltage of 67 kV and 150 μΑ, while the heart #2 was scanned at a voltage of 80 kV and 124 μΑ. All scans were performed with an aluminum filter for a full rotation of 360° at the highest camera resolution. The projection images acquired during the scanning procedure were reconstructed into cross-section images using the SkyScan’s NRecon software (NRecon, Skyscan, Bruker, Belgium) which implements a modified Feldkamp’s back-projection algorithm. Furthermore, 3D volume renderings of the scanned specimen were created using the CTVox software (CTVox, Skyscan, Bruker, Belgium) to visually investigate the anatomy of the internal and external 3D structures of the sample.

### DNA isolation and sequencing

Infected arterial bulbs preserved in 95% ethanol were used for DNA extraction. Samples were centrifuged at 8000×*g* for 10 min and the ethanol supernatant was removed. DNA was extracted from the dried pellet using QIAgen DNeasy kit (Qiagen, Valencia, California, USA) according to manufacturer’s instructions. Small subunit ribosomal DNA (*SSU* rDNA) was amplified by the polymerase chain reaction (PCR) technique using Taq PCR Master Mix kit (Qiagen) and the primers MyxospecF (5′-TTC TGC CGT ATC AAC TWG TTG-3′) [19] and 18R (5′-CTA CGG AAA CCT TGT TAC G-3′) [20]. PCR reactions were performed in a BIO-RAD MJ Mini Personal Thermal Cycler with an initial denaturation at 94 °C for 3 min, followed by 35 cycles of denaturation at 94 °C for 1 min, annealing at 56 °C for 1 min and extension at 72 °C for 90 s and a final extension step at 72 °C for 5 min. PCR products were visualized in an ethidium bromide-stained 1% agarose gel and then purified with PURELINK PCR purification kit (Invitrogen - Thermo Fisher Scientific Inc, Waltham MA, USA). PCR products were sequenced using ABI3730xl sequencer (Applied Biosystems) according to the protocol BigDye Terminators 3.1 (Applied Biosystems, ThermoFisher Scientific Inc, Waltham MA, USA).

### Phylogenetic analysis

All available *SSU* rDNA sequences of *Henneguya* species infecting fish were downloaded from GenBank and used in the present study; *Ceratomyxa diplodae* Lubat, Radujkovic, Marques & Bouix, 1989 (KX099691.1) was used as the outgroup. The novel sequences were edited by eye, trimmed to 1830 bp with Geneious 9.1 and aligned with the GenBank sequences using ClustalW in Geneious 9.1 with default settings. Phylogenetic analysis was done by Neighbour-Joining method in MEGA 7 [21] using Tamura-Nei model and pairwise deletion option. Genetic distances were calculated using MEGA 7 and the “compute pairwise distances” option of the Tamura-Nei model.

## Results

### Gross pathology

Sporadic incidents of diagnosed red sea bream henneguyosis from Leros Island date back to 2010. In these cases, either persisting low morbidity and daily mortality (≤ 1%), or sudden deaths of apparently healthy-looking fish were reported with occasional minor superficial skin lesions. Mortality was low and not associated with a particular size range of fish or season of year but was of concern to the farmers when large marketable fish were lost or when candidate broodstock fish under quarantine were affected. Most of the fish examined (~ 90%) appeared normal externally with no hemorrhages on skin or erosion of fins, but sometimes the skin was dull with superficial inflammation, minor erosions and scale loss. Gills were moderately inflamed with excessive mucus secretions.

**Fig. 1:**
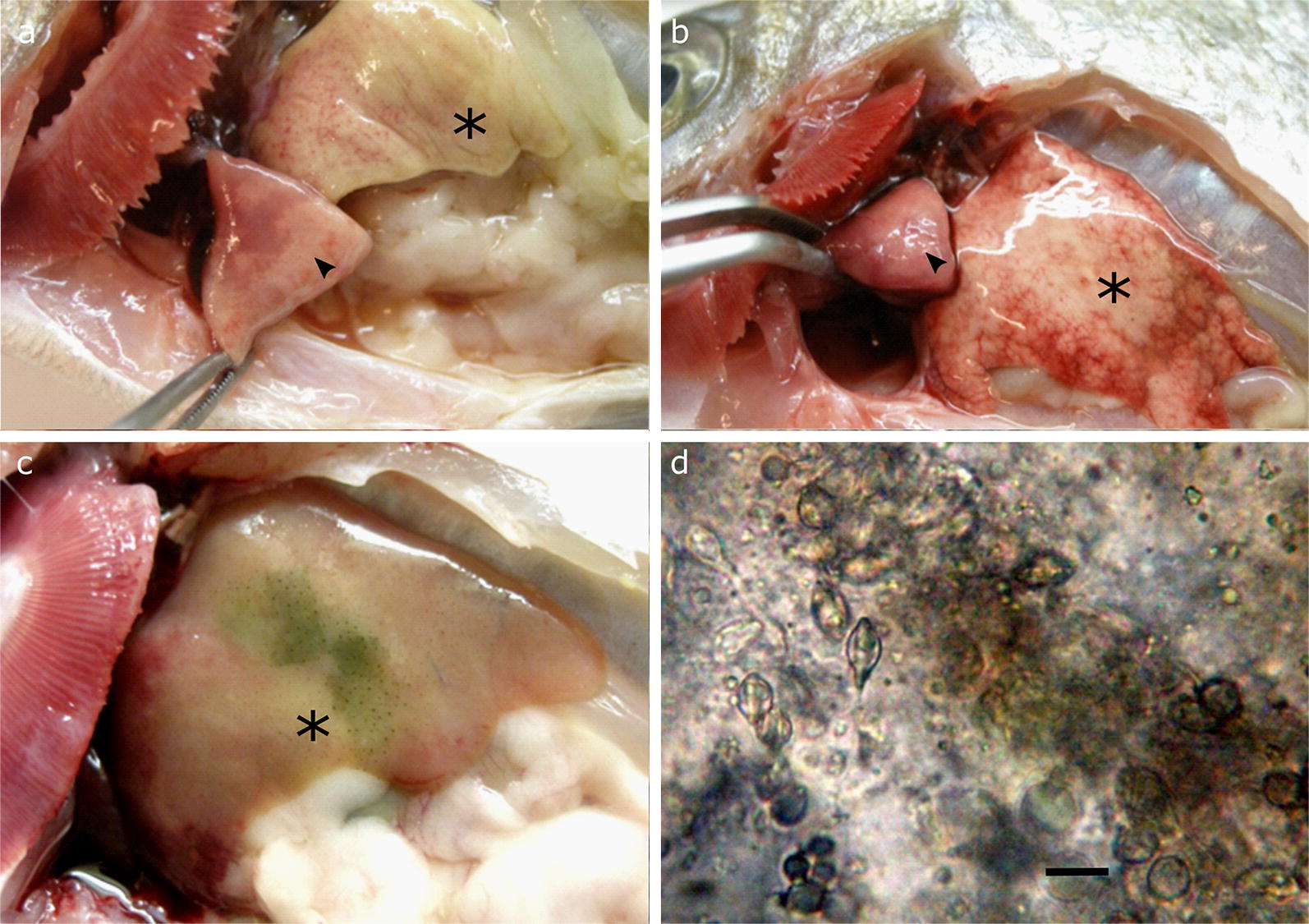
**a**, **b** Infected hearts (arrowheads) with noticeable myocardium degeneration. Haemorrhagic and degenerated livers (asterisks) (**a** and **b**) with bile imbibition (**c**). **d** Mature spores of *Henneguya aegea* n. sp. in fresh liver tissue squash under light microscopy (magnification ×400). *Scale-bar*: **d**, 10 µm

**Fig. 2:**
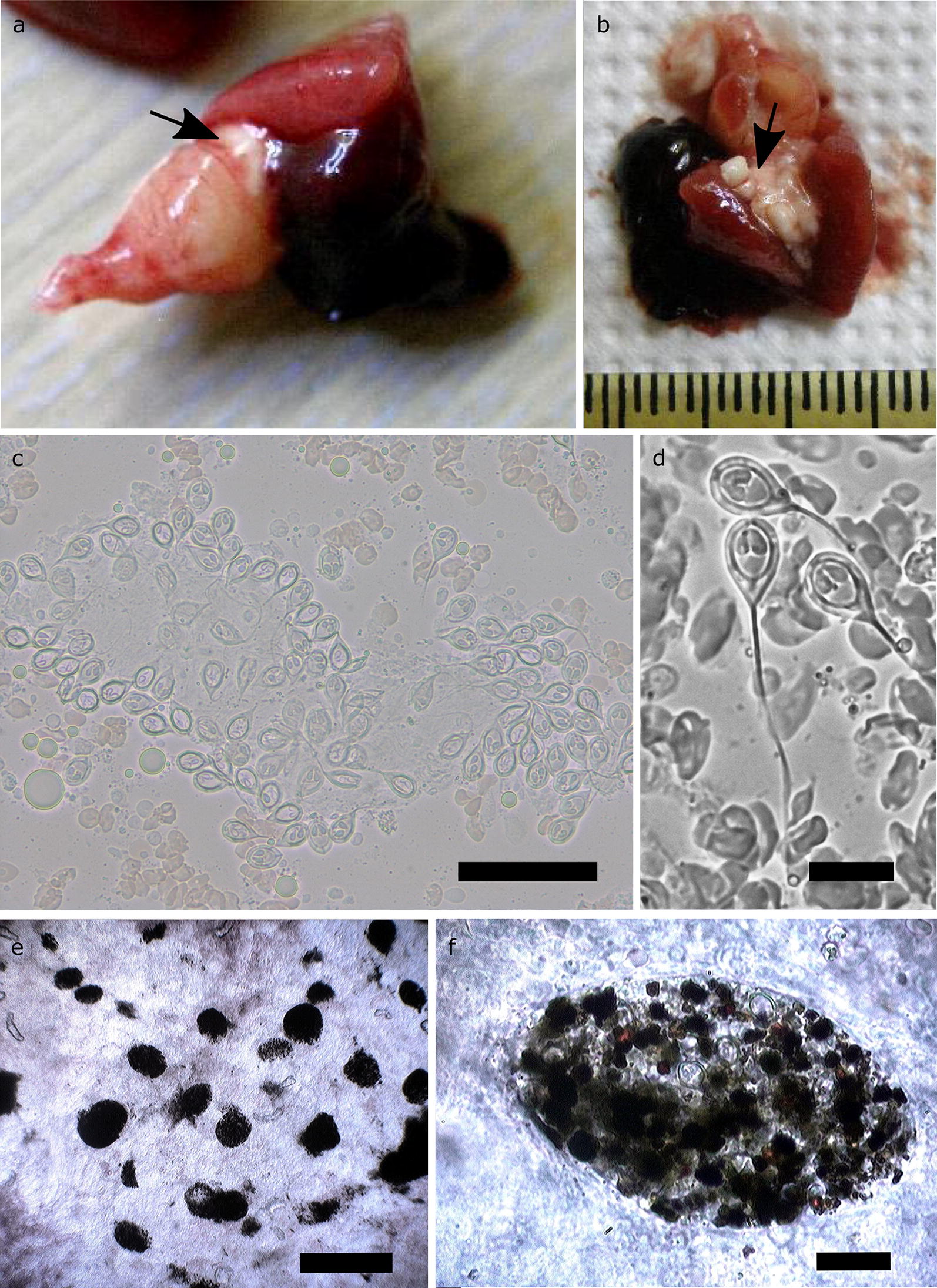
**a** Infected heart with distended bulbus arteriosus; note the plasmodium (arrow) at the basis of bulbus arteriosus. **b** Numerous large white plasmodia inside the ventricle (arrow) containing large numbers of myxosporean spores. **c** Developing spores of *Henneguya aegea* n. sp. from a ruptured plasmodium under light microscopy. **d** Light microscopy of unstained mature *H. aegea* n. sp. (magnification ×400) (under phase contrast). **e** Proliferation of melanomacrophage centers in *Henneguya*-infected kidney (squash mount). **f** Higher magnification of the macrophage accumulation with *H. aegea* n. sp. spores. *Scale-bars*: **c**, 50 µm; **d**, 10 µm; **e**, 20 µm; **f**, 20 µm

At gross examination, infected hearts had enlarged arterial bulbs as well as obvious areas of myocardium degeneration (Figs. 1a, b, 2a). When dissected, these hearts revealed numerous large, white to cream-colored, 1 to 2 mm in diameter, irregularly shaped plasmodia in the ventricle (Fig. 2b) containing large numbers of developing *Henneguya* sp. spores (Fig. 2c). Abundant free mature spores were found in microscopic examination of the atrium and bulbus arteriosus (Fig. 2d). Spores were apparently disseminated *via* blood circulation to all body parts and organs. Numerous mature spores were found in the kidney, eliciting a host response characterized by proliferation of the melanomacrophage centers (Fig. 2e). Examination of the macrophage aggregations in fresh squash preparations of the kidney by light microscopy at higher magnification revealed that they encompassed free parasitic spores (Fig. 2f). A few spores were also present in the ventricle, gills, digestive mucosa, liver parenchyma (Fig. 1d) and gall-bladder wall.

Livers at necropsy appeared swollen and pale due to vacuolar hepatic lipidosis, sometimes with petechial hemorrhages and areas with a greenish tinge apparently due to post-mortem bile imbibition (Fig. 1a–c). Cohesive hepatic parenchyma (squash) observed under the microscope showed diffuse fatty degeneration (vacuolar hepatopathy) and an abundance of *Henneguya* spores (Fig. 1d).

### Histopathology

**Fig. 3:**
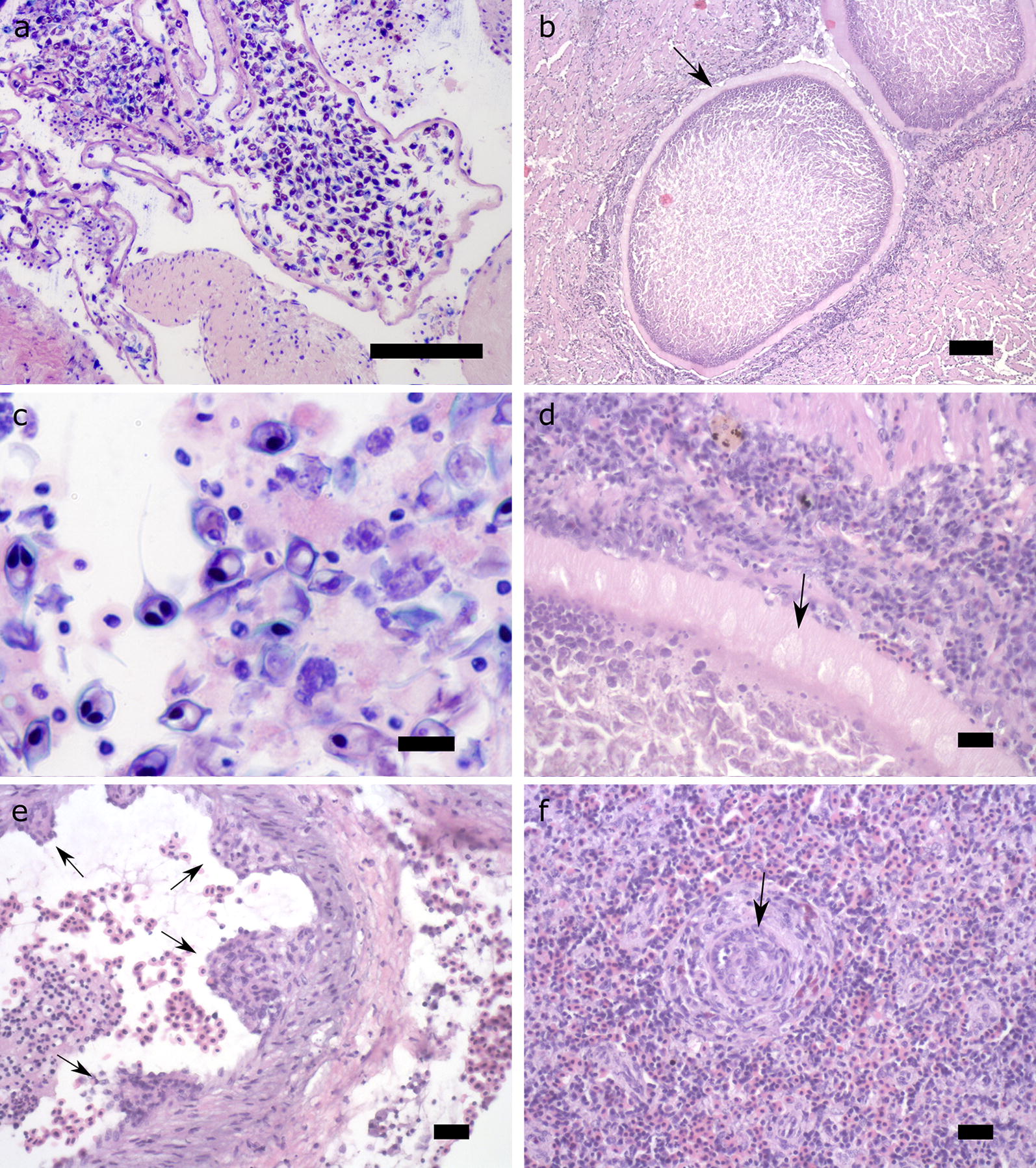
**a** Developed plasmodium containing a large number of developing spores in the bulbus arteriosus of the affected fish (Resin embedded tissue; methylene blue/azure II/basic fuchsin staining). **b** Intact plasmodia of the parasite in the bulbus arteriosus (paraffin-embedded tissue; H&E staining) with broad eosinophilic capsule (arrow). **c** Developing spores of *Henneguya aegea* n. sp. from the heart at higher magnification showing the dark-stained pyriform polar capsules (resin-embedded tissue; methylene blue/azure II/basic fuchsin staining). **d** Capsule of the plasmodium with ellipsoid clearer areas indicating pinocytotic channels (arrow). **e** Papilliform proliferated intima (arrows) of a large blood vessel wall in the heart. **f** Proliferation of the intima of the blood vessels in the spleen obstructing the lumen (arrow). *Scale-bars*: **a**, 100 µm; **b**, 200 µm; **c**, 10 µm; **d**, 40 µm; **e**, 40 µm; **f**, 20 µm

Multiple cysts (plasmodia) of the myxozoan parasite with a broad eosinophilic, hyaline cyst walls measuring 10 μm in thickness were observed in the lumen of the ventricle and bulbus arteriosus of the infected hearts (Fig. 3a, b, d). Within the cysts, numerous developing spores were visible (Fig. 3a, c). Several ellipsoid clear areas directed towards the arterial lumen of the bulbus were observed in the cyst wall; these were previously described as pinocytotic channels [22]. The cysts observed were often closely aligned to the endothelium but without real attachment. The endothelium of the endocardium and numerous large vessels showed papilliform proliferations into the vessel lumens consisting of the endothelial cells (Fig. 3e). Within the myocardium, there were only very small foci of degeneration consisting of loss of the myofibers and slight infiltrations of macrophages and lymphocytes. Similar proliferations of the intima of larger blood vessels were also present especially in the spleen and liver. Here, the lumens of the vessels were almost completely obstructed by the intima proliferations (arteriosclerosis). This lesion was characterized more by proliferative and degenerative changes than inflammation within the vessel wall. Few inflammatory cells were intermingled within the proliferations (Fig. 3f). Gills were mostly normal with very mild fusion of some secondary lamellae with focal lymphocytes and histiocytic infiltrates.

### Micro-CT

**Fig. 4:**
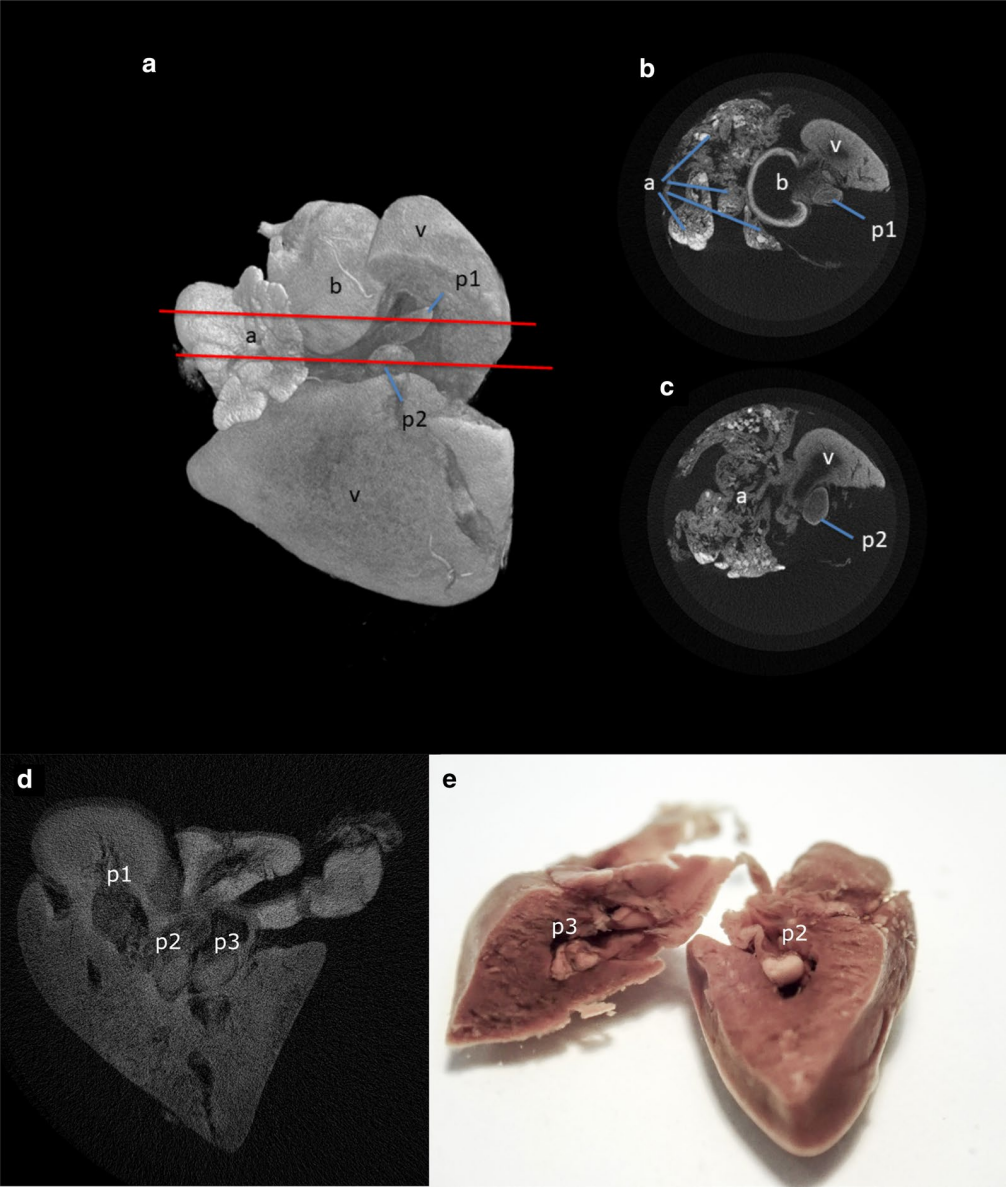
Images of infected hearts using the micro-CT technology, showing the location and the appearance of the plasmodia in the ventricle. **a** 3D volume rendering of the heart #1. Two plasmodia (p1, p2) of *Henneguya aegea* n. sp. are visible through a dissection cut in the ventricle. Red lines indicate the section plan of the corresponding 2D cross-section images (**b** and **c**). **d** Cross-section image of the heart #2 showing two large plasmodia in the ventricle (p2 and p3) and one in the junction of the bulbus arteriosus with the ventricle. **e** Heart #2 (scanned in D) dissected in the area where the plasmodia were identified through micro-CT showing the actual location and appearance of p2 and p3. *Abbreviations*: a, atrium; b, bulbus arteriosus; p, plasmodium; v, ventricle

In the first sample (heart #1), the micro-CT scans revealed numerous large plasmodia located in the ventricle (Fig. 4a–c). A membrane surrounding the plasmodia was visible in the images. In this sample, no plasmodia were found in the bulbus arteriosus. In the second sample (heart #2), three large plasmodia were identified in the ventricle and one in the basis of the bulbus arteriosus (Fig. 4d). Following scanning, the sample was dissected in the area where the plasmodia were seen in micro-CT in order to validate the tomography findings (Fig. 4e). The plasmodia were removed, and squash preparations confirmed that these were plasmodia of the *Henneguya* sp. Using the CTAnalyzer software (CTAN, Skyscan, Bruker, Belgium), we measured the volume of the three plasmodia and the ventricle. The three plasmodia occupied approximately 2% of the total volume of the ventricle. A video of the scanning of the two hearts is provided in Additional file 1: Video S1.

**Family Myxobolidae Thélohan, 1892**

**Genus** ***Henneguya*** **Thélohan, 1892**

#### *Henneguya aegea* n. sp

*Type-host*: *Pagrus major* (Temminck & Schlegel) (Perciformes: Sparidae), red seabream.

*Type-locality*: Off Leros, Aegean Sea, Greece.

*Type-material*: Type (air-dried slide stained with Giemsa) is deposited in the collection of the Natural History Museum of Crete, Heraklion, Greece (accession no. NHMC60.8).

*Site of infection*: Heart, bulbus arteriosus and ventricle.

*Prevalence*: 50% (10 out of 20 fish).

*Representative DNA sequence*: *SSU* rRNA gene, GenBank accession number MK007473.

*ZooBank registration*: To comply with the regulations set out in article 8.5 of the amended 2012 version of the *International Code of Zoological Nomenclature* (ICZN) [23], details of the new species have been submitted to ZooBank. The Life Science Identifier (LSID) of the article is urn:lsid:zoobank.org:pub:CDC0D4EF-5A29-4A0B-937D-6908E8E2570F.

*Etymology*: Aegea (feminine) in Greek, originating from the Aegean Sea, where the fish were cultured.

### Description

**Fig. 5:**
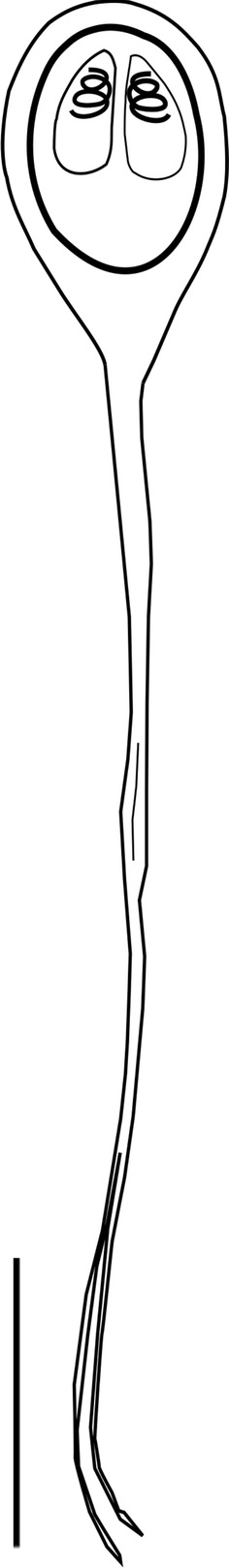
Line drawing of *Henneguya aegea* n. sp. *Scale-bar*: 10 μm

**Fig. 6:**
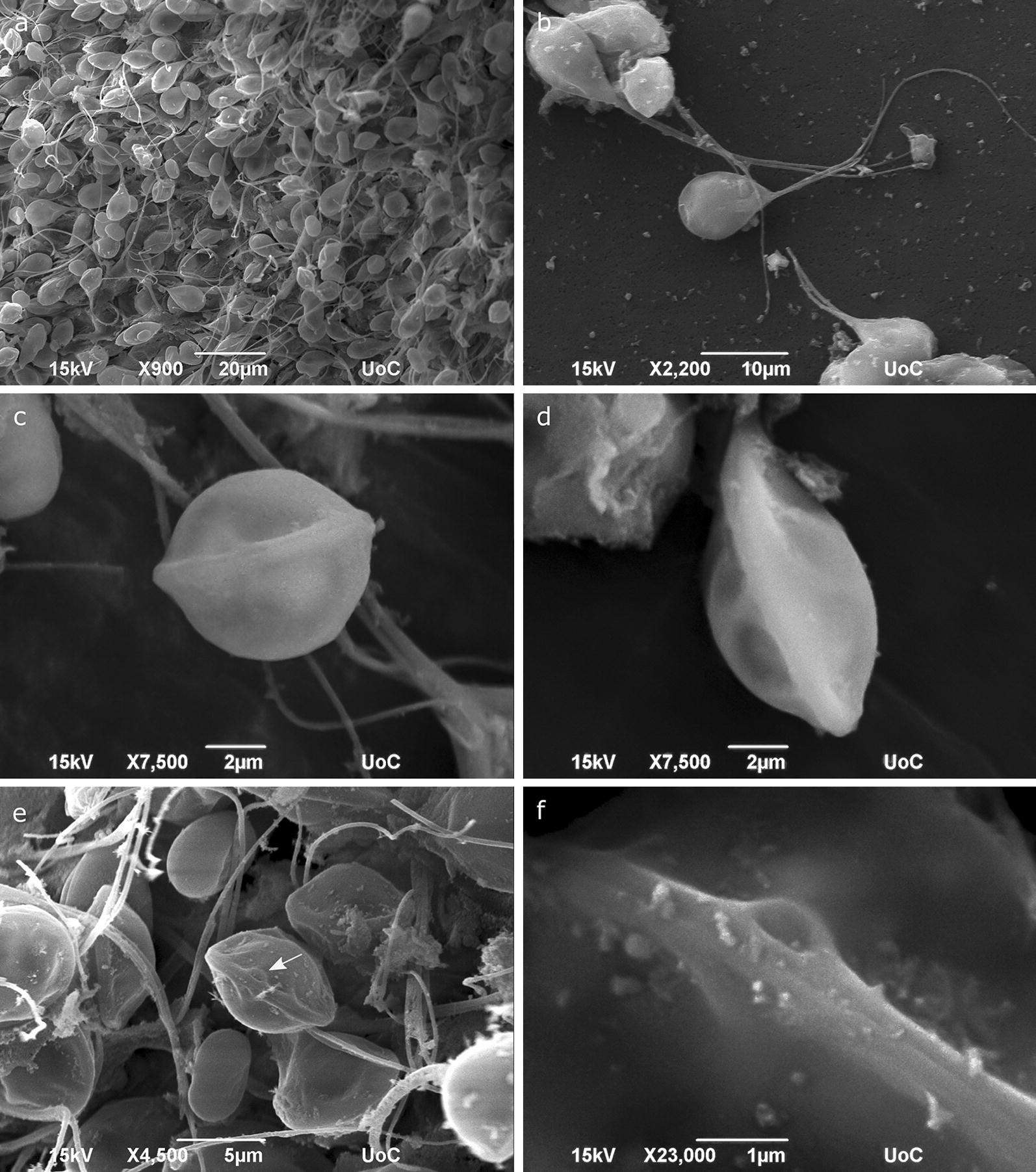
SEM micrographs. **a** Numerous spores of *Henneguya aegea* n. sp. from a ruptured plasmodium. **b** Frontal view of the spores. **c** Frontal view of the spore showing the sutural line. **d** Side view of the spore. **e** Frontal view of the spore showing two polar tubule discharge pores (arrow) located over the sutural line at each valve. **f** Higher magnification of the polar tubule discharge pores

[Based on 55 spores. Figs. 1d, 2c, d, 3c, 5, 6a–e] Spore body oval-shaped in frontal view, with slightly attenuated posterior end elongated into 2 caudal processes (Figs. 2d, 5). Spore body length 10.0–14.9 (12.8 ± 1.0), width 5.7–10.9 (8.2 ± 1.1), wall thickness 5.2–7.0 (6.2 ± 0.6). Caudal processes 2, equal in length, 34.1–60.6 (42.6 ± 6.5). Polar capsules 2, pyriform, 1.4–6.8 (3.4 ± 0.6) in length, 0.8–2.5 (1.7 ± 0.3) in width. Total length (spore body and processes) 53.6–82.8 (64.9 ± 8.7).

### Differential diagnosis

A comparation of taxonomic characters with congeneric species of the genus (Table 1) does not clearly indicate significant similarity. Median length and width of the spores of *H. aegea* n. sp. resemble those reported for *H. mauritaniensis* Khlifa, Miller, Adlard, Faye & Sasal, 2012 and the *Henneguya* spp. found on *Pagrus major* in Italy (even though no spore thickness is reported in these two species) but the new species differs in all other examined parameters. Measurements of the polar capsule are less variable between *Henneguya* spp. and can be seen in the newly described species as similar to the ones described in *H. cynoscioni* Dykova, Buron, Roumillat & Fiala, 2011, *H. akule* Work, Takata, Whipps & Kent, 2008, *H. pagri*, *H. lateolabracis* Yokoyama, Kawakami, Yasuda & Tanaka, 2003, *H. vitiensis* Laird, 1950, and *H. yokoyama* Li, Sato, Kamata, Ohnishi & Sugita-Konishi 2012.

**Table 1:** Comparative data for *Henneguya aegea* n. sp. and other congeneric species

Species	Host	Site in host	Locality	Spore (L × W)	Thickness	Polar capsule (L × W)	Caudal process length	Reference
*Henneguya aegea* n. sp.	*Pagrus majo*r	Heart	Off Greece	10.0–14.8 × 5.6–10.9 (12.8 × 8.1)	5.1–7.0 (6.2)	1.4–6.8 × 0.8–2.5 (3.4 × 1.7)	34.1–60.6 (42.6)	Present study
*H. akule*	*Selar crumenophthalmus*	Bulbus arteriosus	Hawaii	12.1 × 7.4		3.4 × 1.4	28.7	[39]
*H. cynoscioni*	*Cynoscion nebulosus*	Bulbus arteriosus	Off USA	10.4 × 8.8		3.3 × 2	8.1	[22]
*H. lateolabracis*	*Lateolabrax* sp.	Bulbus arteriosus	Off Japan	9.9–11.9 × 6.4–7.8 (10.7 × 7.5)	5.9–6.4 (6.2)	3.0–4.0 × 1.5–2.0 (3.4 ×1.7)	30.7–49.5 (37.7)	[37]
*H. mauritaniensis*	*Pagrus caeruleostictus*	Bulbus arteriosus	Off Mauritania	12.3 × 8.0		4.1 × 3.0	25.3	[29]
*H. otolithi*	*Otolithes rubber*; *O. maculatus*	Bulbus arteriosus	Off India	10–12 × 6–8.5	4–5	3.0–4.0 × 2.0–2.5	35.0–40.0	[42]
*H. ogawai*	*Acanthopagrus schlegelii*	Alimentary tract	Off Japan	8.9–12.2 × 6.3–7.5 (11.0 × 6.9)	5.2–6.6 (5.9)	3.8–5.2 × 1.4–2.3 (4.3 ×1.9)	8.4–12.7 (10.0)	[43]
*H. pagri*	*Pagrus major*	Bulbus arteriosus	Off Japan	9.9–11.9 × 6.4–8.4 (10.5 × 7.5)	5.4–6.4 (5.9)	2.5–4.0 × 1.5–2.0 (3.1×1.6)	24.8–34.7 (29.6)	[13]
*H. sebasta*	*Sebastes paucispinis*	Bulbus arteriosus	Off USA	13.0–17.5 × 5.6–11.0 (15.1 × 9.2)	5.0–8.7 (7.1)	3.7–5.6 × 1.8–3.1 (4.5 × 2.4)	32.5–87.5 (62.0)	[40]
*H. tunisiensis*	*Symphodus tinca*	Gill arches	Off Tunisia	13.1 × 9.1		2.0 × 4.0	28.4	[26]
*H. vitiensis*	*Leiognathus fasciatus*	Heart	Off Fiji	13.7 × 7.8		3.2×1.7	29.1	[41]
*H. yokoyamai*	*Acanthopagrus schlegelii*	Gall-bladder	Off Japan	10.1–13.7 × 6.6–7.5 (11.0 ×7.1)	4.5–6.4 (5.6)	3.1–4.2 × 1.8–2.4 (3.7 ×2.0)	10.8–17.0 (14.1)	[43]
*H. zikawiensis*	*Carassius auratus*	Heart	Off China	10.0–14.0 × 8.0–10.0 (11.4 × 8.5)	7.0–8.0 (7.3)	4.0–5.5 × 2.5–4.0 (4.7 × 3.3)		[44]
*Henneguya* sp.	*Sparus aurata*	Bulbus arteriosus	Off Italy	12.9 × 7.8		4.5 × 2.5	23.2	[14]
*Henneguya* sp.	*Sparus aurata*	Gills	Off Tunisia	13.4 × 9.47		4.75 × 2.25	26.5	[15]

*Note:* Measurements are given in μm

*Abbreviations*: L, length; W, width

Taking into account the high intraspecific size variability of parasites in the same sample and the possibility that the length of the caudal processes is no longer considered a systematic character, other infection parameters and/or molecular data should be taken into consideration to determine species distinct status. To our knowledge, *Henneguya aegea* n. sp. is the first member of the genus with plasmodia developing not only in the bulbus arteriosus but also in the ventricle (tissue tropism).

### Scanning electron microscopy (SEM)

Observations with SEM showed that the spores had a smooth surface and two unequal valves which were joined forming a conspicuous sutural line. At the front part of the spore, two polar tubule discharge pores were observed, each situated in either valve connected to the sutural line. The long caudal processes were separated but were in contact for at least the first half of their length (Fig. 6).

### Molecular analysis and phylogeny

**Fig. 7:**
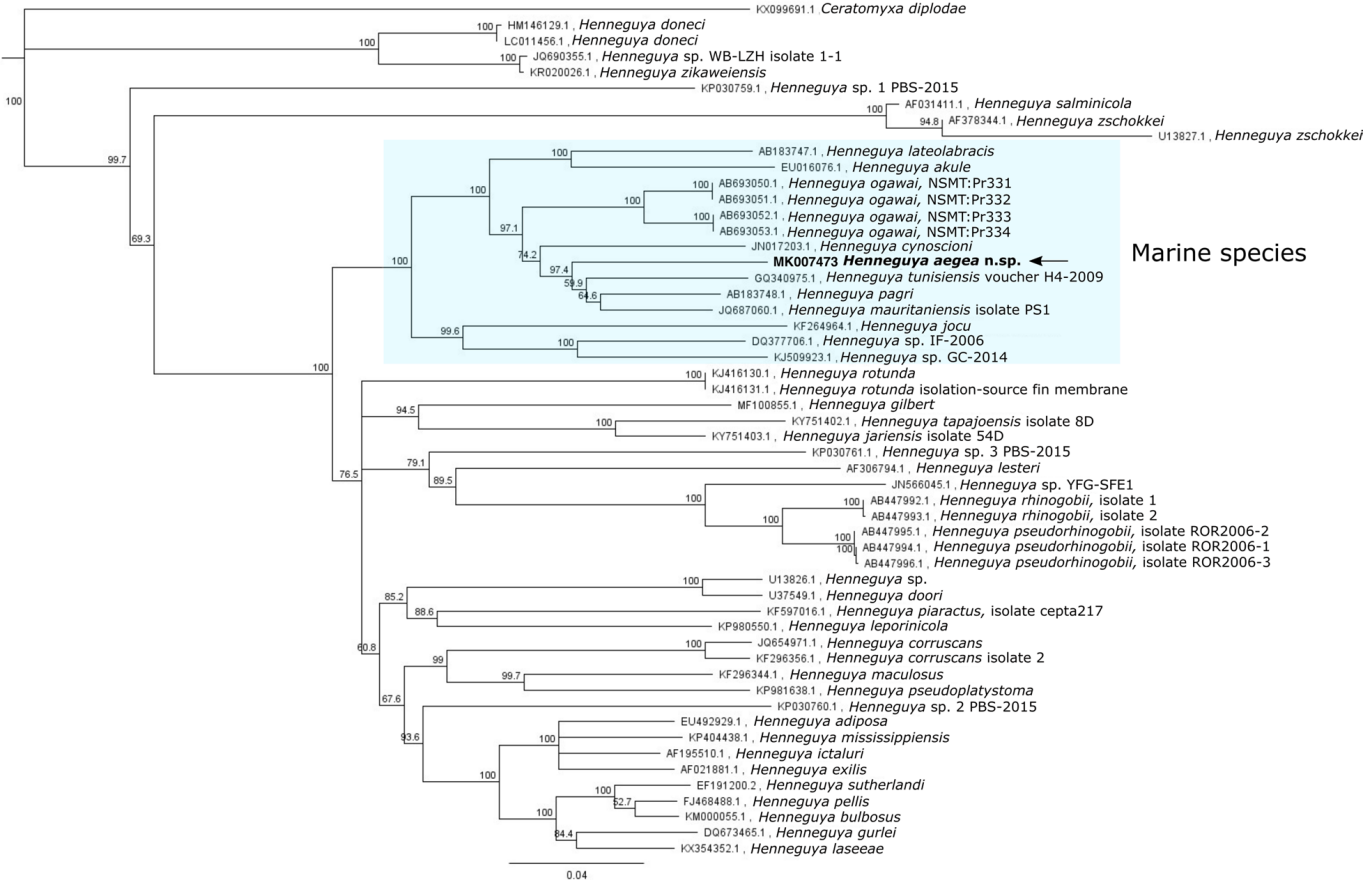
Neighbor-joining tree based on all *18S* rRNA gene sequences for *Henneguya* spp. available on GenBank (August 2018). *Henneguya aegea* n. sp. is grouped with the other *Henneguya* spp. infecting marine species and forms a separate clade with *H. cynoscioni*, *H. tunisiensis*, *H. pagri* and *H. mauritaniensis*. Numbers at the nodes represent bootstrap values for the nodes gaining more than 50% support. The scale-bar indicates the percentage of genetic variation

A small subunit rRNA (*SSU* rRNA) gene sequence of 1727 nt in length was obtained from *H. aegea* n. sp. All *SSU* rRNA gene sequences (*n* = 52) from *Henneguya* spp. available on GenBank (August 2018) were retrieved and aligned with *H. aegea* n. sp. sequence using ClustalW in Geneious 9.1. Following trimming which resulted in an alignment with a total of 1830 characters including alignment gaps, a phylogenetic tree using the Neighbor-Joining method with 1000 bootstraps was constructed with *Ceratomyxa diplodae* as the outgroup. *Henneguya aegea* n. sp. clustered with the other *Henneguya* spp. infecting marine species and formed a separate clade with *H. cynoscioni*, *H. tunisiensis* (Bahri, Marton, Marques & Eszterbauer, 2010), *H. pagri* and *H. mauritaniensis* (Fig. 7). The highest similarity expressed as % identity (% of bases/residues which are identical) was with *H. mauritaniensis* (89.8%) followed by *H. pagri* (89.5%).

## Discussion

As marked in the differential diagnosis, the spore measurements of the new species resemble those of *Henneguya* sp. reported by Caffara et al. [14] in Italy, but differs in other parameters such as the length and width of the median polar capsule and the length of the caudal processes. The caudal processes of *H. aegea* n. sp. are much longer than those of *Henneguya* sp. found in the gilthead seabream and close to those in *H. otolithi* Ganapati, 1941 and *H. lateolabracis*. However, it has been suggested that the caudal processes of myxobolids might not be a valid taxonomic character. The fact that this character is intermixed within the phylogeny of myxobolids suggests that the genetic capacity to develop spore tails exists broadly within this family, but only certain lineages express it. The irregular occurrence of caudal processes throughout the myxozoan tree of life is emphasized further by the appearance of this trait in the spores of some species of *Myxobolus* Bütschli, 1882 as reported for *M. turpisrotundus* Zhang, 2009 [24]. The close affinity between *Myxobolus* and *Henneguya* has been revealed in both morphological and phylogenetic studies [6, 24–26].

Identification of myxosporean parasites can be greatly assisted by their tissue/organ preference since apart from being highly host-specific, many myxosporeans also show high tissue tropism [27]. The plasmodial stages of *H. aegea* n. sp. were located in the heart tissue, in the bulbus arteriosus and the ventricle, although many of the spores were observed in the liver and few in the gills and kidney, likely as a result of dispersion of the parasite through the blood flow. Several myxosporean species have been found to infect fish hearts (Table 1); according to Ye et al. [28] the list of heart-infecting myxosporeans included ten *Henneguya* spp. (*H. zikawiensis* Sikama, 1938, *H. otolithi*, *H. vitiensis*, *H. sebasta* Moser & Love, 1975, *H. brachideuteri* Kpatcha, Faye, Diebakate, Fall & Toguebaye, 1997, *H. ouakamensis* Kpatcha, Faye, Diebakate, Fall & Toguebaye, 1997, *H. yoffensis* Kpatcha, Faye, Diebakate, Fall & Toguebaye, 1997, *H. lateolabracis*, *H. pagri* and *H. akule*; five *Kudoa* spp., and 18 *Myxobolus* spp. In addition to these, three other *Henneguya* spp. have been reported to infect hearts: *H. mauritaniensis* and *H. cynoscioni* from *Pagrus caeruleostictus* (Valenciennes) and *Cynoscion nebulosus* (Cuvier), respectively [22, 29] and one unidentified *Henneguya* sp. from the gilthead seabream [14]. The plasmodia of the majority of the heart-infecting *Henneguya* spp. are located exclusively in the bulbus arteriosus. The plasmodia of *H. aegea* n. sp. were found both in the bulbus arteriosus but also in the ventricle of its host which differentiates this species from the other described *Henneguya* spp. To our knowledge, the only other parasite of this genus that has its plasmodia in the ventricle is *Myxobolus hearti* Chen, 1998 from the Prussian carp, *Carassius gibelio* (Bloch) [28], which, however, does not have plasmodia in the bulbus arteriosus.

With the growing molecular data that have become available in recent years, we examined the phylogenetic position of the new species, using the *SSU* rRNA gene as a phylogenetic marker against all sequences from congeneric species available on GenBank. In the phylogenetic tree, *H. aegea* n. sp. grouped with the other *Henneguya* spp. infecting marine species and formed a separate clade with *H. cynoscioni*, *H. tunisiensis*, *H. pagri* and *H. mauritaniensis*. The phylogeny obtained is in agreement with previously published analyses for *Henneguya* spp. [22, 30].

Based on morphology/morphometry data, tissue tropism and comparison with other available genetic sequences (molecular phylogenetic analysis), we concluded that the species isolated in the present study is a novel species, *H. aegea* n. sp. One of the most interesting aspects of our study is related to the fact that the host, *P. major* is not native to the Mediterranean but rather introduced in the late 1980s [31]. This, together with the fact that myxobolid parasites are generally highly host-specific [32] suggests that the cultured *P. major* has contracted the parasite from a wild host, which remains unknown. It can be hypothesized that the original host is genetically close to *P. major*, more likely a member of the family Sparidae since myxobolid parasites usually infect a single host or a limited number of closely related hosts. The possibility that the novel parasite was brought to the Mediterranean Sea *via* the introduced host is highly unlikely. Only fertilized eggs of *P. major* were imported to Europe from Japan and thus *H. aegea* n. sp. could not be brought together since myxosporeans are not transmitted vertically. Generally, only a small fraction of the parasite species accompanies their host during its introduction to a new area [33]. Torchin et al. [33] suggested that “introduced populations are often derived from relatively small subsets of native populations (and sometimes from uninfected life-history stages), and this reduces the probability of introducing parasites along with a host species. Another potential limitation for the establishment of introduced parasites is that many parasites have complex life-cycles requiring more than one host”. Both elements of this hypothesis are valid in this case since imported eggs can be seen as uninfected stages of the host and the life-cycle of *Henneguya* spp. involves an actinosporean alternative stage that develops in an oligochaete [34].

Despite the fact that the parasite was probably not introduced with the host, there is a clear risk that has emerged with the importation of *P. major* into the Mediterranean. If the native/local parasite is highly host-specific and the introduced host is competent for that particular parasite, there is a tendency for amplification of the disease with a “spillback” onto native hosts [35]. These concepts, however, have been assessed and examined in respect to invasive or introduced host species that may interact freely in the new locality with native hosts. The situation in aquaculture differs significantly, since introduced fish species are confined in a limited space and may act as a reservoir for the parasites. An example comes from outbreaks of *Kudoa amamiensis* Egusa & Nakajima, 1980 in farmed Japanese amberjacks, *Seriola quinqueradiata* Temminck & Schlegel, introduced from northern to southern Japan, where amberjacks were assumed to be accidental hosts of the parasite that was found in several native wild fishes [36]. Future studies should focus on the identification of the natural hosts (fish and possible non-fish intermediate hosts) in the specific geographical location in order to evaluate the likelihood of the spillback hypothesis.

The spores of *H. lateolabracis* and *H. pagri* are the cause of lesions in the heart, spleen and gill tissues of the affected fish as already described by other authors [13, 37]. Arteriolosclerosis is common in the animal kingdom, as is the well-known atherosclerosis in humans, and is defined as a chronic arterial change with loss of elasticity and narrowing of the lumen by proliferation of the arterial wall with degeneration [38]. It differs from atherosclerosis lesions commonly seen in humans which is characterized by inflammation, fatty degeneration and mineralization of the vessel wall and is rather a degenerative disease. The narrowing of the vessel lamina by the intima proliferations can lead to tissue infarctions and degeneration of the surrounding tissue in any organ, especially under conditions of increased stress and activity. A similar result was found in a case of the Prussian carp infected with *M. hearti* [28]. Although arteriosclerosis in animals leads less often to infarction than atherosclerosis in humans, and is often regarded as a minor finding in animals, we believe that the severity of the lesions in the tissues examined with complete occlusion of the arterial lumen, might be the cause or a major contributor to the increased mortalities of the diseased fish.

Micro-CT technology provides fascinating insights regarding the morphology and the location of the plasmodia in a non-destructive way. The same samples can be later used for histology or dissection in order to confirm and further study the presence of the plasmodia as in this case or in other lesions. In our investigation, we have also noticed significant differences between the healthy and the infected hearts in regard to the density of the tissue (data not shown). In a following study, we intend to study these differences in combination with histopathology.

## Conclusions

This study showed that even highly host-specific parasites may affect exotic species causing disease and morbidity. The novel myxobolid parasite, *H. aegea* n. sp. caused lesions in the blood vessels of cultured *P. major* which is an introduced species in the Mediterranean. The natural host of the parasite is not known; however, based on the host specificity of *Henneguya* spp. and the type of pathology observed, it is expected that the presence of a novel competent host raised in aquaculture at high densities could result in spillback of *H. aegea* n. sp. to native fish host populations.

## Supplementary information

**Additional file** **1****: Video S1.** 3D-video rendering of the two micro-CT scans of the two infected hearts showing, in different section planes, the internal location of spore-containing plasmodia (red arrows).

## Data Availability

Data supporting the conclusions of this article are included within the article. The type-material was deposited in the Museum of Natural History of Crete under the accession number NHMC60.8. The newly generated sequence was deposited in the GenBank database under the accession number MK007473.

## Abbreviations

*18S* rDNA: 18S ribosomal deoxyribonucleic acid
SEM: scanning electron microscopy
H&E: haematoxylin and eosin
Micro-CT: micro X-ray computed tomography
PTA: phosphotungstic acid
HCMR: Hellenic Centre for Marine Research
MP: megapixels
CCD: charged coupled device
PCR: polymerase chain reaction
SD: standard deviation
